# Supplementary material for: Associations between white matter integrity and postural control in adults with traumatic brain injury
Source: PLoS One. 2023 Nov 27;18(11):e0288727. doi: 10.1371/journal.pone.0288727 (PMC10681193; doi:10.1371/journal.pone.0288727)
Supplement: S1 File — Description of the computation of B-H corrected p values for each set of correlations. (DOCX) [file pone.0288727.s001.docx]

**Benjamini-Hochberg (B-H) procedure**

1. Sort individual p values in ascending order and assign ranks to these p values

2. Calculate each p value’s Benjamini-Hochberg critical value using the formula q*(R/T) where

- q (false discovery rate) = 0.05
- R = rank
- T = total number of tests

3. Compare individual p value to its corresponding critical value (i.e., q*(R/T))

4. Select the largest p value that is smaller than the critical value

**Computation of Benjamini-Hochberg corrected p value for SOT-ROI correlations**

| **p value** | **Rank[R]** | **q value** | | **Total tests (T)** | | | **q*(R/T)** | **Significance** | |
| --- | --- | --- | --- | --- | --- | --- | --- | --- | --- |
| 0.001 | 1 | 0.05 | | 35 | | | 0.001429 | 1 | |
| 0.003 | 2 | 0.05 | | 35 | | | 0.002857 | 1 | |
| 0.004 | 3 | 0.05 | | 35 | | | 0.004286 | 1 | |
| 0.005 | 4 | 0.05 | | 35 | | | 0.005714 | 1 | |
| 0.007 | 5 | 0.05 | | 35 | | | 0.007143 | 1 | |
| 0.008 | 6 | 0.05 | | 35 | | | 0.008571 | 1 | |
| 0.009 | 7 | 0.05 | | 35 | | | 0.01 | 1 | |
| 0.011 | 8 | 0.05 | | 35 | | | 0.011429 | 1 | |
| 0.013 | 9 | 0.05 | | 35 | | | **0.012857** | 1 | |
| 0.019 | 10 | 0.05 | | 35 | | | 0.014286 | 0 | |
| 0.023 | 11 | 0.05 | | 35 | | | 0.015714 | 0 | |
| 0.026 | 12 | 0.05 | | 35 | | | 0.017143 | 0 | |
| 0.029 | 13 | 0.05 | | 35 | | | 0.018571 | 0 | |
| 0.030 | 14 | 0.05 | | 35 | | | 0.02 | 0 | |
| 0.039 | 15 | 0.05 | | 35 | | | 0.021429 | 0 | |
| 0.042 | 16 | 0.05 | | 35 | | | 0.022857 | 0 | |
| 0.063 | 17 | 0.05 | | 35 | | | 0.024286 | 0 | |
| 0.065 | 18 | 0.05 | | 35 | | | 0.025714 | 0 | |
| 0.066 | 19 | 0.05 | | 35 | | | 0.027143 | 0 | |
| 0.069 | 20 | 0.05 | | 35 | | | 0.028571 | 0 | |
| 0.100 | 21 | 0.05 | | 35 | | | 0.03 | 0 | |
| 0.112 | 22 | 0.05 | | 35 | | | 0.031429 | 0 | |
| 0.115 | 23 | 0.05 | | 35 | | | 0.032857 | 0 | |
| 0.120 | 24 | 0.05 | | 35 | | | 0.034286 | 0 | |
| 0.172 | 25 | 0.05 | | 35 | | | 0.035714 | 0 | |
| 0.213 | 26 | 0.05 | | 35 | | | 0.037143 | 0 | |
| 0.237 | 27 | 0.05 | | 35 | | | 0.038571 | 0 | |
| 0.268 | 28 | 0.05 | | 35 | | | 0.04 | 0 | |
| 0.291 | 29 | 0.05 | | 35 | | | 0.041429 | 0 | |
| 0.469 | 30 | 0.05 | | 35 | | | 0.042857 | 0 | |
| 0.539 | 31 | 0.05 | | 35 | | | 0.044286 | 0 | |
| 0.626 | 32 | 0.05 | | 35 | | | 0.045714 | 0 | |
| 0.654 | 33 | 0.05 | | 35 | | | 0.047143 | 0 | |
| 0.910 | 34 | 0.05 | | 35 | | | 0.048571 | 0 | |
| 0.980 | 35 | 0.05 | | 35 | | | 0.05 | 0 | |
| Note: adjusted p value ≤ 0.013 is considered significant (Test=1) | | | | | | | | | |
|  |  | |  | |  |  | | |  |
|  |  | |  | |  |  | | |  |

**Computation of Benjamini-Hochberg corrected p value for LOS-ROI correlations**

| **p value** | **Rank[R]** | **q value** | **Total test(T)** | **q*(R/T)** | **Significance** |
| --- | --- | --- | --- | --- | --- |
| 0.000 | 1 | 0.05 | 85 | 0.000588 | 1 |
| 0.001 | 2 | 0.05 | 85 | 0.001176 | 1 |
| 0.001 | 3 | 0.05 | 85 | 0.001765 | 1 |
| 0.001 | 4 | 0.05 | 85 | 0.002353 | 1 |
| 0.002 | 5 | 0.05 | 85 | 0.002941 | 1 |
| 0.002 | 6 | 0.05 | 85 | 0.003529 | 1 |
| 0.003 | 7 | 0.05 | 85 | 0.004118 | 1 |
| 0.003 | 8 | 0.05 | 85 | 0.004706 | 1 |
| 0.003 | 9 | 0.05 | 85 | 0.005294 | 1 |
| 0.004 | 10 | 0.05 | 85 | 0.005882 | 1 |
| 0.004 | 11 | 0.05 | 85 | 0.006471 | 1 |
| 0.004 | 12 | 0.05 | 85 | 0.007059 | 1 |
| 0.004 | 13 | 0.05 | 85 | 0.007647 | 1 |
| 0.004 | 14 | 0.05 | 85 | 0.008235 | 1 |
| 0.005 | 15 | 0.05 | 85 | 0.008824 | 1 |
| 0.005 | 16 | 0.05 | 85 | 0.009412 | 1 |
| 0.005 | 17 | 0.05 | 85 | 0.01 | 1 |
| 0.005 | 18 | 0.05 | 85 | 0.010588 | 1 |
| 0.005 | 19 | 0.05 | 85 | 0.011176 | 1 |
| 0.005 | 20 | 0.05 | 85 | 0.011765 | 1 |
| 0.006 | 21 | 0.05 | 85 | 0.012353 | 1 |
| 0.006 | 22 | 0.05 | 85 | 0.012941 | 1 |
| 0.007 | 23 | 0.05 | 85 | 0.013529 | 1 |
| 0.007 | 24 | 0.05 | 85 | 0.014118 | 1 |
| 0.008 | 25 | 0.05 | 85 | 0.014706 | 1 |
| 0.009 | 26 | 0.05 | 85 | 0.015294 | 1 |
| 0.009 | 27 | 0.05 | 85 | 0.015882 | 1 |
| 0.009 | 28 | 0.05 | 85 | 0.016471 | 1 |
| 0.010 | 29 | 0.05 | 85 | 0.017059 | 1 |
| 0.012 | 30 | 0.05 | 85 | 0.017647 | 1 |
| 0.013 | 31 | 0.05 | 85 | 0.018235 | 1 |
| 0.014 | 32 | 0.05 | 85 | 0.018824 | 1 |
| 0.015 | 33 | 0.05 | 85 | 0.019412 | 1 |
| 0.015 | 34 | 0.05 | 85 | 0.02 | 1 |
| 0.016 | 35 | 0.05 | 85 | 0.020588 | 1 |
| 0.017 | 36 | 0.05 | 85 | 0.021176 | 1 |
| 0.018 | 37 | 0.05 | 85 | 0.021765 | 1 |
| 0.020 | 38 | 0.05 | 85 | 0.022353 | 1 |
| 0.022 | 39 | 0.05 | 85 | **0.022941** | 1 |
| 0.025 | 40 | 0.05 | 85 | 0.023529 | 0 |
| 0.031 | 41 | 0.05 | 85 | 0.024118 | 0 |
| 0.031 | 42 | 0.05 | 85 | 0.024706 | 0 |
| 0.032 | 43 | 0.05 | 85 | 0.025294 | 0 |
| 0.034 | 44 | 0.05 | 85 | 0.025882 | 0 |
| 0.036 | 45 | 0.05 | 85 | 0.026471 | 0 |
| 0.037 | 46 | 0.05 | 85 | 0.027059 | 0 |
| 0.038 | 47 | 0.05 | 85 | 0.027647 | 0 |
| 0.041 | 48 | 0.05 | 85 | 0.028235 | 0 |
| 0.042 | 49 | 0.05 | 85 | 0.028824 | 0 |
| 0.044 | 50 | 0.05 | 85 | 0.029412 | 0 |
| 0.045 | 51 | 0.05 | 85 | 0.03 | 0 |
| 0.047 | 52 | 0.05 | 85 | 0.030588 | 0 |
| 0.049 | 53 | 0.05 | 85 | 0.031176 | 0 |
| 0.050 | 54 | 0.05 | 85 | 0.031765 | 0 |
| 0.051 | 55 | 0.05 | 85 | 0.032353 | 0 |
| 0.051 | 56 | 0.05 | 85 | 0.032941 | 0 |
| 0.052 | 57 | 0.05 | 85 | 0.033529 | 0 |
| 0.053 | 58 | 0.05 | 85 | 0.034118 | 0 |
| 0.056 | 59 | 0.05 | 85 | 0.034706 | 0 |
| 0.056 | 60 | 0.05 | 85 | 0.035294 | 0 |
| 0.058 | 61 | 0.05 | 85 | 0.035882 | 0 |
| 0.059 | 62 | 0.05 | 85 | 0.036471 | 0 |
| 0.061 | 63 | 0.05 | 85 | 0.037059 | 0 |
| 0.067 | 64 | 0.05 | 85 | 0.037647 | 0 |
| 0.077 | 65 | 0.05 | 85 | 0.038235 | 0 |
| 0.086 | 66 | 0.05 | 85 | 0.038824 | 0 |
| 0.097 | 67 | 0.05 | 85 | 0.039412 | 0 |
| 0.108 | 68 | 0.05 | 85 | 0.04 | 0 |
| 0.110 | 69 | 0.05 | 85 | 0.040588 | 0 |
| 0.152 | 70 | 0.05 | 85 | 0.041176 | 0 |
| 0.174 | 71 | 0.05 | 85 | 0.041765 | 0 |
| 0.183 | 72 | 0.05 | 85 | 0.042353 | 0 |
| 0.203 | 73 | 0.05 | 85 | 0.042941 | 0 |
| 0.219 | 74 | 0.05 | 85 | 0.043529 | 0 |
| 0.231 | 75 | 0.05 | 85 | 0.044118 | 0 |
| 0.238 | 76 | 0.05 | 85 | 0.044706 | 0 |
| 0.289 | 77 | 0.05 | 85 | 0.045294 | 0 |
| 0.309 | 78 | 0.05 | 85 | 0.045882 | 0 |
| 0.361 | 79 | 0.05 | 85 | 0.046471 | 0 |
| 0.382 | 80 | 0.05 | 85 | 0.047059 | 0 |
| 0.395 | 81 | 0.05 | 85 | 0.047647 | 0 |
| 0.411 | 82 | 0.05 | 85 | 0.048235 | 0 |
| 0.529 | 83 | 0.05 | 85 | 0.048824 | 0 |
| 0.561 | 84 | 0.05 | 85 | 0.049412 | 0 |
| 0.876 | 85 | 0.05 | 85 | 0.05 | 0 |
| Note: adjusted p value ≤ 0.023 is considered significant (Test=1) | | | | | |

**Computation of Benjamini-Hochberg corrected p value for LOS-SOT correlations**

| **p value** | | **Rank[R]** | | **q value** | | **Total test(T)** | | **q*(R/T)** | | **Significance** | |  |
| --- | --- | --- | --- | --- | --- | --- | --- | --- | --- | --- | --- | --- |
| 0.000 | 1 | | 0.05 | | 35 | | 0.001429 | | 1 | |  |  |
| 0.000 | 2 | | 0.05 | | 35 | | 0.002857 | | 1 | |  |  |
| 0.001 | 3 | | 0.05 | | 35 | | 0.004286 | | 1 | |  |  |
| 0.001 | 4 | | 0.05 | | 35 | | 0.005714 | | 1 | |  |  |
| 0.001 | 5 | | 0.05 | | 35 | | 0.007143 | | 1 | |  |  |
| 0.002 | 6 | | 0.05 | | 35 | | 0.008571 | | 1 | |  |  |
| 0.002 | 7 | | 0.05 | | 35 | | 0.01 | | 1 | |  |  |
| 0.003 | 8 | | 0.05 | | 35 | | 0.011429 | | 1 | |  |  |
| 0.004 | 9 | | 0.05 | | 35 | | 0.012857 | | 1 | |  |  |
| 0.007 | 10 | | 0.05 | | 35 | | 0.014286 | | 1 | |  |  |
| 0.009 | 11 | | 0.05 | | 35 | | 0.015714 | | 1 | |  |  |
| 0.013 | 12 | | 0.05 | | 35 | | 0.017143 | | 1 | |  |  |
| 0.014 | 13 | | 0.05 | | 35 | | 0.018571 | | 1 | |  |  |
| 0.014 | 14 | | 0.05 | | 35 | | 0.02 | | 1 | |  |  |
| 0.015 | 15 | | 0.05 | | 35 | | 0.021429 | | 1 | |  |  |
| 0.019 | 16 | | 0.05 | | 35 | | **0.022857** | | 1 | |  |  |
| 0.028 | 17 | | 0.05 | | 35 | | 0.024286 | | 0 | |  |  |
| 0.031 | 18 | | 0.05 | | 35 | | 0.025714 | | 0 | |  |  |
| 0.035 | 19 | | 0.05 | | 35 | | 0.027143 | | 0 | |  |  |
| 0.050 | 20 | | 0.05 | | 35 | | 0.028571 | | 0 | |  |  |
| 0.053 | 21 | | 0.05 | | 35 | | 0.03 | | 0 | |  |  |
| 0.064 | 22 | | 0.05 | | 35 | | 0.031429 | | 0 | |  |  |
| 0.089 | 23 | | 0.05 | | 35 | | 0.032857 | | 0 | |  |  |
| 0.110 | 24 | | 0.05 | | 35 | | 0.034286 | | 0 | |  |  |
| 0.112 | 25 | | 0.05 | | 35 | | 0.035714 | | 0 | |  |  |
| 0.174 | 26 | | 0.05 | | 35 | | 0.037143 | | 0 | |  |  |
| 0.210 | 27 | | 0.05 | | 35 | | 0.038571 | | 0 | |  |  |
| 0.213 | 28 | | 0.05 | | 35 | | 0.04 | | 0 | |  |  |
| 0.218 | 29 | | 0.05 | | 35 | | 0.041429 | | 0 | |  |  |
| 0.233 | 30 | | 0.05 | | 35 | | 0.042857 | | 0 | |  |  |
| 0.234 | 31 | | 0.05 | | 35 | | 0.044286 | | 0 | |  |  |
| 0.365 | 32 | | 0.05 | | 35 | | 0.045714 | | 0 | |  |  |
| 0.396 | 33 | | 0.05 | | 35 | | 0.047143 | | 0 | |  |  |
| 0.496 | 34 | | 0.05 | | 35 | | 0.048571 | | 0 | |  |  |
| 0.653 | 35 | | 0.05 | | 35 | | 0.05 | | 0 | |  |  |

Note: adjusted p value ≤ 0.023 is considered significant (Test=1)
