# Supplementary material for: Associations between white matter integrity and postural control in adults with traumatic brain injury
Source: PLoS One. 2023 Nov 27;18(11):e0288727. doi: 10.1371/journal.pone.0288727 (PMC10681193; doi:10.1371/journal.pone.0288727)
Supplement: S2 File — These are complementary tables showing correlation values between each balance measure and DTI measures that were not included in the main manuscript (Axial Diffusivity, Medial Diffusivity and Radial Diffusivity). (DOCX) [file pone.0288727.s002.docx]

**Correlations between balance measures (SOT and LOS) and DTI measures (Axial Diffusivity, Medial Diffusivity and Radial Diffusivity) controlled for age.**

**Table 6. Correlations between SOT and Axial Diffusivity measures controlled for age (n=52).**

| ROI | Statistics | EqC1 | EqC2 | EqC3 | EqC4 | EqC5 | EqC6 | Comp |
| --- | --- | --- | --- | --- | --- | --- | --- | --- |
| Corticospinal tract | r | 0.012 | -0.055 | 0.036 | 0.102 | 0.139 | 0.077 | 0.129 |
|  | p value | 0.933 | 0.692 | 0.794 | 0.463 | 0.316 | 0.582 | 0.354 |
| Inferior cerebellar peduncle | r | -0.096 | -0.183 | -0.072 | -0.005 | -0.103 | -0.109 | -0.095 |
|  | p value | 0.491 | 0.186 | 0.604 | 0.971 | 0.457 | 0.433 | 0.494 |
| Middle cerebellar peduncle | r | 0.103 | 0.058 | 0.039 | -0.097 | -0.175 | -0.227 | -0.161 |
|  | p value | 0.461 | 0.678 | 0.780 | 0.486 | 0.205 | 0.099 | 0.246 |
| Medial lemniscus | r | -0.102 | 0.107 | -0.103 | 0.030 | 0.026 | -0.029 | -0.029 |
|  | p value | 0.464 | 0.441 | 0.457 | 0.828 | 0.850 | 0.833 | 0.834 |
| Posterior thalamus radiation | r | 0.071 | -0.038 | 0.056 | 0.037 | -0.096 | -0.089 | -0.070 |
|  | p value | 0.609 | 0.784 | 0.687 | 0.792 | 0.489 | 0.520 | 0.617 |

Note abbreviations: ROI=region of interest, EqC1=Equilibrium Score condition 1, EqC2= Equilibrium Score condition 2, EqC3= Equilibrium Score condition 3, EqC4= Equilibrium Score condition 4, EqC5= Equilibrium Score condition 5, EqC6= Equilibrium Score condition 6, Comp= composite score

**Table 7. Correlations between SOT and Medial Diffusivity measures controlled for age (n=52).**

| ROI | Statistics | EqC1 | EqC2 | EqC3 | EqC4 | EqC5 | EqC6 | Comp |
| --- | --- | --- | --- | --- | --- | --- | --- | --- |
| Corticospinal tract | r | -0.082 | -0.041 | -0.030 | 0.013 | 0.051 | -0.011 | 0.030 |
|  | p value | 0.553 | 0.770 | 0.829 | 0.928 | 0.713 | 0.936 | 0.829 |
| Inferior cerebellar peduncle | r | -0.198 | -0.171 | -0.173 | -0.119 | -0.216 | -0.223 | -0.218 |
|  | p value | 0.150 | 0.217 | 0.212 | 0.392 | 0.116 | 0.105 | 0.113 |
| Middle cerebellar peduncle | r | 0.093 | 0.077 | -0.007 | -0.154 | -0.245 | -0.373 | -0.256 |
|  | p value | 0.502 | 0.578 | 0.962 | 0.265 | 0.074 | 0.005 | 0.061 |
| Medial lemniscus | r | -0.149 | 0.052 | -0.212 | -0.033 | -0.045 | -0.105 | -0.095 |
|  | p value | 0.283 | 0.708 | 0.124 | 0.815 | 0.747 | 0.452 | 0.496 |
| Posterior thalamus radiation | r | -0.085 | -0.104 | -0.090 | -0.073 | -0.170 | -0.187 | -0.181 |
|  | p value | 0.539 | 0.453 | 0.515 | 0.601 | 0.218 | 0.175 | 0.190 |

Note abbreviations: ROI=region of interest, EqC1=Equilibrium Score condition 1, EqC2= Equilibrium Score condition 2, EqC3= Equilibrium Score condition 3, EqC4= Equilibrium Score condition 4, EqC5= Equilibrium Score condition 5, EqC6= Equilibrium Score condition 6, Comp= composite score

**Table 8. Correlations between SOT and Radial Diffusivity measures controlled for age (n=52).**

| ROI | Statistics | EqC1 | EqC2 | EqC3 | EqC4 | EqC5 | EqC6 | Comp |
| --- | --- | --- | --- | --- | --- | --- | --- | --- |
| Corticospinal tract | r | -0.129 | -0.024 | -0.055 | -0.050 | -0.022 | -0.088 | -0.054 |
|  | p value | 0.352 | 0.864 | 0.693 | 0.720 | 0.872 | 0.528 | 0.697 |
| Inferior cerebellar peduncle | r | -0.229 | -0.156 | -0.215 | -0.180 | -0.280 | -0.285 | -0.289 |
|  | p value | 0.096 | 0.260 | 0.118 | 0.192 | 0.041 | 0.037 | 0.034 |
| Middle cerebellar peduncle | r | 0.006 | 0.039 | -0.089 | -0.282 | -0.325 | -0.471 | -0.364 |
|  | p value | 0.964 | 0.778 | 0.522 | 0.039 | 0.017 | 0.000 | 0.007 |
| Medial lemniscus | r | -0.180 | 0.057 | -0.238 | -0.091 | -0.089 | -0.127 | -0.139 |
|  | p value | 0.193 | 0.682 | 0.083 | 0.513 | 0.523 | 0.360 | 0.315 |
| Posterior thalamus radiation | r | -0.153 | -0.139 | -0.183 | -0.128 | -0.215 | -0.259 | -0.244 |
|  | p value | 0.271 | 0.317 | 0.184 | 0.356 | 0.118 | 0.059 | 0.075 |

Note abbreviations: ROI=region of interest, EqC1=Equilibrium Score condition 1, EqC2= Equilibrium Score condition 2, EqC3= Equilibrium Score condition 3, EqC4= Equilibrium Score condition 4, EqC5= Equilibrium Score condition 5, EqC6= Equilibrium Score condition 6, Comp= composite score

**Table 9. Correlations between LOS and Axial Diffusivity measures controlled for age (n=53).**

| ROI | Statistics | RT-C | MVL-C | EPE-C | MXE-C | DCL-C |
| --- | --- | --- | --- | --- | --- | --- |
| Anterior thalamus radiation | r | 0.033 | 0.141 | 0.078 | 0.041 | 0.104 |
|  | p value | 0.813 | 0.305 | 0.571 | 0.768 | 0.452 |
| Corpus callosum-front | r | 0.033 | -0.026 | -0.056 | -0.129 | 0.141 |
|  | p value | 0.812 | 0.849 | 0.682 | 0.347 | 0.304 |
| Corpus callosum-posterior | r | 0.001 | 0.020 | -0.094 | -0.091 | 0.143 |
|  | p value | 0.995 | 0.885 | 0.496 | 0.510 | 0.298 |
| Corpus callosum-superior | r | -0.097 | 0.253 | -0.006 | 0.008 | 0.017 |
|  | p value | 0.482 | 0.063 | 0.968 | 0.953 | 0.900 |
| Cingulum | r | 0.100 | 0.070 | -0.184 | -0.123 | -0.085 |
|  | p value | 0.470 | 0.610 | 0.179 | 0.370 | 0.539 |
| Corticopontine tract | r | 0.147 | 0.046 | -0.184 | -0.097 | -0.072 |
|  | p value | 0.284 | 0.738 | 0.179 | 0.480 | 0.603 |
| Corticospinal tract | r | -0.009 | 0.080 | 0.023 | 0.043 | 0.145 |
|  | p value | 0.948 | 0.561 | 0.865 | 0.754 | 0.292 |
| Inferior cerebellar peduncle | r | 0.070 | 0.090 | 0.012 | -0.014 | -0.077 |
|  | p value | 0.612 | 0.511 | 0.930 | 0.919 | 0.577 |
| Inferior frontooccipital fasciculus | r | 0.053 | 0.040 | -0.069 | -0.160 | 0.093 |
|  | p value | 0.701 | 0.770 | 0.619 | 0.243 | 0.500 |
| Inferior longitudinal fasciculus | r | 0.041 | 0.134 | -0.063 | -0.101 | -0.092 |
|  | p value | 0.766 | 0.330 | 0.645 | 0.461 | 0.505 |
| Middle cerebellar peduncle | r | 0.251 | -0.072 | -0.050 | 0.016 | -0.104 |
|  | p value | 0.064 | 0.599 | 0.715 | 0.906 | 0.449 |
| Medial lemniscus | r | 0.112 | -0.179 | -0.003 | -0.022 | -0.006 |
|  | p value | 0.416 | 0.191 | 0.983 | 0.876 | 0.962 |
| Optic radiation | r | 0.173 | -0.026 | -0.081 | -0.167 | 0.025 |
|  | p value | 0.206 | 0.848 | 0.559 | 0.222 | 0.853 |
| Optic tracts | r | 0.214 | -0.133 | -0.029 | -0.054 | 0.018 |
|  | p value | 0.116 | 0.333 | 0.836 | 0.695 | 0.898 |
| Posterior thalamus radiation | r | -0.005 | 0.150 | 0.036 | -0.029 | -0.056 |
|  | p value | 0.972 | 0.276 | 0.792 | 0.831 | 0.684 |
| Superior frontooccipital fasciculus | r | -0.079 | 0.155 | 0.113 | 0.007 | 0.112 |
|  | p value | 0.567 | 0.257 | 0.413 | 0.959 | 0.415 |
| Superior longitudinal fasciculus | r | 0.140 | 0.128 | -0.030 | -0.044 | 0.044 |
|  | p value | 0.307 | 0.353 | 0.825 | 0.750 | 0.752 |

Note abbreviations: ROI=region of interest, RT=reaction time, MV=-movement velocity, EPE=endpoint excursion, MXE=maximal excursion, DCL=directional control

**Table 10. Correlations between LOS and Medial Diffusivity measures controlled for age (n=53).**

| ROI | Statistics | RT-C | MVL-C | EPE-C | MXE-C | DCL-C |
| --- | --- | --- | --- | --- | --- | --- |
| Anterior thalamus radiation | r | 0.196 | 0.026 | -0.169 | -0.146 | -0.082 |
|  | p value | 0.152 | 0.851 | 0.218 | 0.286 | 0.553 |
| Corpus callosum-front | r | 0.172 | -0.176 | -0.220 | -0.279 | -0.062 |
|  | p value | 0.209 | 0.200 | 0.107 | 0.039 | 0.653 |
| Corpus callosum-posterior | r | 0.206 | -0.179 | -0.202 | -0.195 | 0.005 |
|  | p value | 0.132 | 0.192 | 0.138 | 0.153 | 0.970 |
| Corpus callosum-superior | r | 0.102 | 0.026 | -0.158 | -0.168 | -0.180 |
|  | p value | 0.459 | 0.850 | 0.250 | 0.219 | 0.189 |
| Cingulum | r | 0.231 | -0.111 | -0.311 | -0.241 | -0.224 |
|  | p value | 0.089 | 0.419 | 0.021 | 0.077 | 0.100 |
| Corticopontine tract | r | 0.285 | -0.026 | -0.232 | -0.086 | -0.180 |
|  | p value | 0.035 | 0.850 | 0.089 | 0.533 | 0.189 |
| Corticospinal tract | r | 0.123 | -0.041 | -0.140 | -0.061 | 0.029 |
|  | p value | 0.372 | 0.765 | 0.308 | 0.659 | 0.834 |
| Inferior cerebellar peduncle | r | 0.198 | -0.057 | -0.078 | -0.111 | -0.192 |
|  | p value | 0.148 | 0.678 | 0.572 | 0.422 | 0.159 |
| Inferior frontooccipital fasciculus | r | 0.175 | -0.062 | -0.208 | -0.263 | -0.027 |
|  | p value | 0.202 | 0.651 | 0.128 | 0.052 | 0.847 |
| Inferior longitudinal fasciculus | r | 0.188 | -0.011 | -0.211 | -0.252 | -0.183 |
|  | p value | 0.170 | 0.936 | 0.122 | 0.063 | 0.181 |
| Middle cerebellar peduncle | r | 0.309 | -0.095 | -0.113 | -0.056 | -0.205 |
|  | p value | 0.022 | 0.491 | 0.410 | 0.685 | 0.134 |
| Medial lemniscus | r | 0.207 | -0.283 | -0.104 | -0.118 | -0.051 |
|  | p value | 0.129 | 0.036 | 0.451 | 0.390 | 0.713 |
| Optic radiation | r | 0.286 | -0.125 | -0.244 | -0.325 | -0.061 |
|  | p value | 0.034 | 0.364 | 0.073 | 0.016 | 0.660 |
| Optic tracts | r | 0.276 | -0.184 | -0.119 | -0.138 | -0.068 |
|  | p value | 0.041 | 0.178 | 0.386 | 0.316 | 0.620 |
| Posterior thalamus radiation | r | 0.097 | 0.017 | -0.066 | -0.107 | -0.143 |
|  | p value | 0.481 | 0.900 | 0.634 | 0.439 | 0.299 |
| Superior frontooccipital fasciculus | r | -0.031 | 0.121 | 0.086 | 0.022 | 0.101 |
|  | p value | 0.819 | 0.379 | 0.534 | 0.871 | 0.462 |
| Superior longitudinal fasciculus | r | 0.344 | -0.090 | -0.198 | -0.159 | -0.183 |
|  | p value | 0.010 | 0.514 | 0.148 | 0.247 | 0.181 |

Note abbreviations: ROI=region of interest, RT=reaction time, MV=-movement velocity, EPE=endpoint excursion, MXE=maximal excursion, DCL=directional control

**Table 11. Correlations between LOS and Radial Diffusivity measures controlled for age (n=53).**

| ROI | Statistics | RT-C | MVL-C | EPE-C | MXE-C | DCL-C |
| --- | --- | --- | --- | --- | --- | --- |
| Anterior thalamus radiation | r | 0.258 | -0.058 | -0.254 | -0.221 | -0.172 |
|  | p value | 0.057 | 0.673 | 0.061 | 0.105 | 0.210 |
| Corpus callosum-front | r | 0.217 | -0.256 | -0.309 | -0.348 | -0.169 |
|  | p value | 0.112 | 0.059 | 0.022 | 0.009 | 0.218 |
| Corpus callosum-posterior | r | 0.313 | -0.269 | -0.292 | -0.279 | -0.040 |
|  | p value | 0.020 | 0.047 | 0.030 | 0.039 | 0.772 |
| Corpus callosum-superior | r | 0.168 | -0.086 | -0.228 | -0.266 | -0.207 |
|  | p value | 0.221 | 0.534 | 0.095 | 0.050 | 0.130 |
| Cingulum | r | 0.295 | -0.175 | -0.354 | -0.267 | -0.264 |
|  | p value | 0.029 | 0.201 | 0.008 | 0.049 | 0.052 |
| Corticopontine tract | r | 0.318 | -0.083 | -0.281 | -0.130 | -0.240 |
|  | p value | 0.018 | 0.549 | 0.038 | 0.344 | 0.078 |
| Corticospinal tract | r | 0.211 | -0.130 | -0.220 | -0.138 | -0.041 |
|  | p value | 0.123 | 0.343 | 0.106 | 0.317 | 0.764 |
| Inferior cerebellar peduncle | r | 0.238 | -0.135 | -0.134 | -0.150 | -0.244 |
|  | p value | 0.080 | 0.324 | 0.328 | 0.274 | 0.073 |
| Inferior frontooccipital fasciculus | r | 0.212 | -0.112 | -0.240 | -0.287 | -0.077 |
|  | p value | 0.121 | 0.417 | 0.078 | 0.034 | 0.574 |
| Inferior longitudinal fasciculus | r | 0.281 | -0.098 | -0.275 | -0.302 | -0.214 |
|  | p value | 0.037 | 0.477 | 0.042 | 0.025 | 0.117 |
| Middle cerebellar peduncle | r | 0.345 | -0.142 | -0.184 | -0.086 | -0.298 |
|  | p value | 0.010 | 0.301 | 0.180 | 0.531 | 0.027 |
| Medial lemniscus | r | 0.276 | -0.327 | -0.150 | -0.163 | -0.093 |
|  | p value | 0.041 | 0.015 | 0.274 | 0.233 | 0.502 |
| Optic radiation | r | 0.309 | -0.187 | -0.289 | -0.343 | -0.106 |
|  | p value | 0.022 | 0.171 | 0.032 | 0.010 | 0.440 |
| Optic tracts | r | 0.342 | -0.214 | -0.168 | -0.147 | -0.124 |
|  | p value | 0.011 | 0.116 | 0.220 | 0.284 | 0.366 |
| Posterior thalamus radiation | r | 0.181 | -0.079 | -0.135 | -0.158 | -0.174 |
|  | p value | 0.187 | 0.565 | 0.327 | 0.249 | 0.203 |
| Superior frontooccipital fasciculus | r | -0.023 | 0.125 | 0.059 | 0.004 | 0.072 |
|  | p value | 0.867 | 0.362 | 0.666 | 0.975 | 0.600 |
| Superior longitudinal fasciculus | r | 0.391 | -0.142 | -0.249 | -0.212 | -0.259 |
|  | p value | 0.003 | 0.301 | 0.067 | 0.119 | 0.056 |

Note abbreviations: ROI=region of interest, RT=reaction time, MV=-movement velocity, EPE=endpoint excursion, MXE=maximal excursion, DCL=directional control
